# Preparation, Characterization and Efficacy Evaluation of Synthetic Biocompatible Polymers Linking Natural Antioxidants

**DOI:** 10.3390/molecules171112734

**Published:** 2012-10-26

**Authors:** Sonia Trombino, Roberta Cassano, Teresa Ferrarelli, Sonia Leta, Francesco Puoci, Nevio Picci

**Affiliations:** Department of Pharmaceutical Sciences, University of Calabria, Arcavacata di Rende 87036, Cosenza, Italy

**Keywords:** antioxidants, biocompatible polymers, lipid peroxidation, *trans*-ferulic acid, α-lipoic acid

## Abstract

The purpose of this work was the synthesis, characterization and efficacy evaluation of new biocompatible antioxidant polymers linking *trans*-ferulic acid or *α*-lipoic acid. In particular, ferulic or lipoic acid were introduced in the preformed polymeric backbone. The new antioxidant biopolymers were characterized by Fourier transform infrared spectroscopy and gel permeation chromatography. The degree of functionalization (moles of antioxidant per gram of polymer) was determined by the Gaur-Gupta method for free amino group determination and by the Folin method for the phenolic groups. Their ability to inhibit lipid peroxidation were estimated in rat liver microsomal membranes induced *in vitro* by *tert*-BOOH (*tert*-butyl hydroperoxide), as a source of free radicals. The DPPH (1,1-diphenyl-2-picrylhydrazyl) radical-scavenging effect was also evaluated. The obtained systems, with different solubility, showed strong antioxidant and antiradical activities, suggesting potential use as packaging materials for foods, cosmetics, pharmaceuticals and personal care products. Moreover, the cytotoxicity of the synthesized polymers was also evaluated on Caco-2 cell cultures in order to verify their biocompatibility when exposed to an absorptive epithelial cell line.

## 1. Introduction

Reactive oxygen species such as superoxide anion, hydroxyl radical, peroxide, and singlet oxygen are constantly generated in the human body and they are involved in various physiologically important biological reactions. In particular, reactive oxygen species (ROS) as well as reactive nitrogen species (RNS), can damage nucleic acids, lipids, and proteins, playing a key role in the development of many diseases such as atherosclerosis, arthritis, neurodegenerative disorders, inflammation, cataracts, Parkinson’s disease and diabetes [1,2,3,4,5,6,7,8,9,10]. Antioxidants are compounds able to protect the human body against cellular damage induced by ROS and RNS. For this reason in recent years many efforts have been focused on the applications of antioxidants as medical treatments [11,12,13,14]. 

**Scheme 1:**
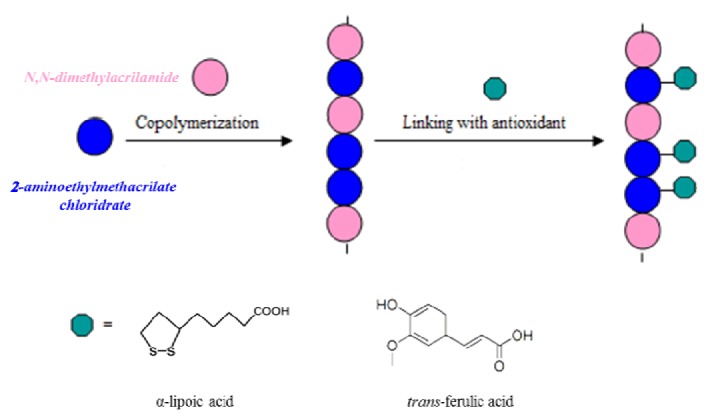
Representation of the preparation of antioxidant polymers.

On the other hand, the use of conventional antioxidants may be conditioned by unfavourable pharmacokinetics, by a short action time due to rapid metabolization, and by excessive absorption that leads to toxic effects. For these reasons, it is thought that linking antioxidant molecules to polymeric matrices by covalent bonds may lead to systems that improve the transport and metabolic stability of low molecular weight antioxidants, and the rate of degradation will be reduced, ensuring a longer persistence than free antioxidants [15,16,17,18,19]. Moreover, they could be applied in those fields in which the use of a single molecule with antioxidant activity is prohibitive; for example, they can be used in hemodialysis applications, in particular, by their introduction in dialysis membranes [20]. In fact, many problems in hemodialysis patients derive from oxidative stress resulting from an imbalance between the production of reactive oxygen species and antioxidant defense mechanisms, which contributes to cardiovascular disease and accelerated atherosclerosis, the major causes of mortality in these patients. Antioxidants are often used also in industry to delay the onset of oxidation processes. In particular, preservatives with antioxidant activity are commonly added to packaged foods to scavenge oxygen radicals. However, many of the preservatives used for foods, textiles, medicines, and other personal care products have been associated with adverse side effects. The strategies to solve these problems are various: reduction of oxygen concentration [21], lowering storage temperature, scavenging of metal ions with sequestrants, coating antioxidants with substances that allow for sustained release [22], mixing of antioxidants with carriers such as synthetic polymers, immobilization of antioxidants on macromolecules of various natures [23]. The last approach is one of the most used in current polymer chemistry, and could provide macromolecular systems in which the antioxidant is still fully efficient [24,25,26,27]. Therefore the aim of this work was the synthesis and characterization of biocompatible polymers, linked to *trans*-ferulic acid or *α*-lipoic acid, two very strong antioxidants. In particular ferulic acid (FA) is one of the most ubiquitous compounds in Nature, especially abundant as an ester form in rice bran pitch, which is obtained when rice oil is produced [28]. This antioxidant compound is currently expected not only to prevent lipid oxidation in food, but also to prevent free-radical-induced diseases such as cancer [29] and atherosclerosis or aging caused by oxidative tissue degeneration. In general, the inhibitory effects of ferulic acid on lipid oxidation as an antioxidant are due to its phenolic nucleus and its conjugated side chain forming a resonance-stabilized phenoxy radical [30]. Lipoic acid (LA) is found endogenously in animals and plants as lipoamide [31]. Its antioxidant properties can be related to four activities: its ability to scavenge free radicals; metal ion chelating activity; capacity to regenerate endogenous antioxidants, for example glutathione and tocopherol, and finally the ability to repair oxidative damage in macromolecules [32,33,34]. The two natural antioxidants were inserted in the macromolecular systems by the synthetic strategies showed in Scheme 1. In particular, we introduced the antioxidants in the preformed polymeric backbone. 

The new antioxidant biopolymers were characterized by Fourier transform infrared spectroscopy (FT-IR) and gel permeation chromatography (GPC). Furthermore, the values of phenolic and aminic contents of all synthesized polymeric materials were determined. Their abilities to inhibit lipid peroxidation were evaluated in rat liver microsomal membranes induced *in vitro* by *tert*-BOOH (*tert*-butyl hydroperoxide), a source of alkoxyl radicals via Fenton reactions. The DPPH (1,1-diphenyl-2-picrylhydrazyl) radical-scavenging effect was also studied. The obtained biocompatible polymers showed strong antioxidant and antiradical activities and different solubilities, suggesting potential usefulness as packaging for foods, cosmetics, pharmaceuticals and personal care products. Finally, their biocompatibility was evaluated against Caco-2 cells.

## 2. Results and Discussion

**Scheme 2:**
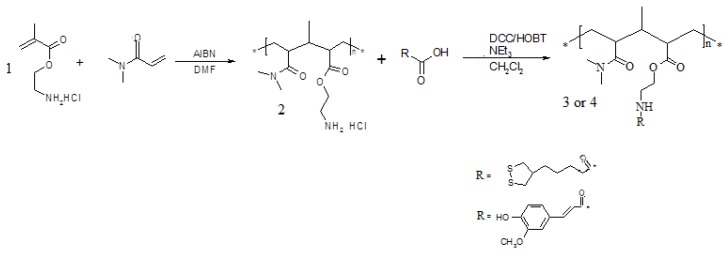
Synthetic route to polymers **3** and **4**.

Antioxidant polymeric matrices linking α-lipoic or *trans*-ferulic acid were prepared. The synthetic route to realize these polymers requires the realization of a macromolecular structure characterized by the presence of functional groups capable of forming stable bonds with the carboxylic groups of the antioxidants. These macromolecules were prepared by copolymerization of *N,N*-dimethylacrylamide, a comonomer known for its biocompatibility, with 2-aminoethylmethacrylate, a monomer containing amino groups suitable for forming amide linkages with the antioxidants in anhydrous CH_2_Cl_2_ and in the presence of condensing agents, such as dicyclohexylcarbodiimide (DCC) and 1-hydroxy-benzotriazole (HOBt) (Scheme 2). 

Polymers **3** and **4** exhibit different solubility characteristics. In particular, **3** is slightly soluble in THF and insoluble in most other protic (H_2_O, EtOH, MeOH, *etc.*) and aprotic (DMF, DMSO, *etc.*) solvents. On the other hand, **4** appears soluble in H_2_O and in some aprotic solvents such as DMF, THF and acetone. The synthesized polymers were characterized by FT-IR spectroscopy which confirmed the amide group formation.The average molecular weight (Mw) of polymer **2**, **3** and **4** and their polidispersity indexes (PI) are reported in Table 1. A high polydispersity is recorded due to the randomness of covalent grafting with ferulic and lipoic acids. 

**Table 1:** Molecular weights and polidispersity indexes of synthesized polymers.

Polymer	Molecular weight (Mw)	Polydispersity index (PI)
1	6.8 kDa	1.7
2	7.3 kDa	2.3
3	7.5 kDa	2.4

Their amino group content was evaluated through the colorimetric method proposed by Gaur-Gupta [35]. For the polymer **3**, a further confirmation of the presence of lipoic groups was furnished through oxidation of thiols with the Ellman reagent, whereas for **4** it was possible to confirm the degree of functionalization, by determining the moles of phenolic groups per gram of polymer, using a colorimetric process known as the Folin test [36]. An examination of the obtained results showed that for **2** the degree of functionalization was 85%. This value indicates that most of the amino groups link lipoic moieties and this may explain the considerable decrease seen in the solubility of **2** in polar solvents compared to that of the starting polymer. Concerning polymer **4**, the total phenolic groups content was about of 6.7% (3.78 × 10^−4^ moles of phenolic groups are present in 1 gram of polymer).

**Figure 1:**
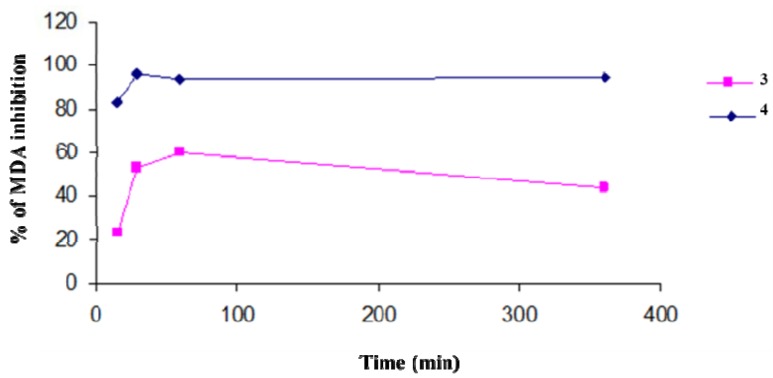
Percentage of inhibition of *tert*-BOOH-induced MDA formation in the presence of polymers **3** and **4** (1 mg/mL) in rat-liver microsomal membranes after 400 min of incubation. The microsomal membranes were incubated with 0.25 × 10^−3^ M *tert*-BOOH at 37 °C under air in the dark. The results represent means ± SEM of six separate experiments.

We evaluated the protection efficiency of our antioxidant polymers against lipidic peroxidation, on microsomial membranes, and through DPPH tests. In particular, the antioxidant activity of **3** and **4**, in inhibiting the lipid peroxidation in rat-liver microsomal membranes [37] induced *in vitro* by a source of hydroxyl radicals (^•^OH) such as tert-BOOH, was evaluated during 400 min of incubation. The microsomal membranes were incubated in the presence of tert-BOOH (250 µM) in air and in the dark. The different formation of malondialdehyde (MDA) in the absence and presence of the antioxidant polymers is an index of their protective effects (Figure 1). Both polymers exibit a maximum antioxidant potency after a few minutes of contact with the reactive species. This efficacy begins to decrease with time for **3**, while the activity of **4** remains almost constant and unchanged over time. The antioxidant activities of polymers were similar to those of the free lipoic and ferulic acids (data not shown). 

**Figure 2:**
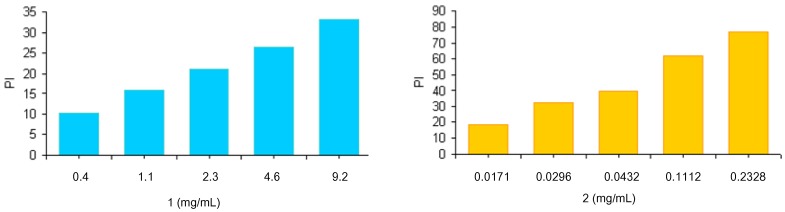
Scavenging effects of antioxidant polymers on the DPPH free radical. The results represent means ± SEM of three determinations.

The radical scavenging ability of the polymers **3** and **4** was assessed through reaction of materials with stable 2,2-diphenyl-1-picrylhydrazyl (DPPH) radicals. Because of its unpaired electron, the picrylhydrazyl radical shows a strong absorption band at 517 nm, assuming a purple coloration. When this radical species is captured by an antioxidant, the absorption decreases and the resultant discoloration is directly proportional to the number of radicals captured. For this test a stock solution of DPPH at a known concentration is used as a control. The absorption of this solution is evaluated spectrophotometrically in the absence and presence of increasing and known amounts of the polymers **3** and **4**. Results, reported as inhibition percentage (PI) of DPPH radicals and evaluated by the equation PI = [1 − (Abs antioxidant polymers)/Abs DPPH] (Figure 2), were compared to those obtained using increasing amounts of lipoic and ferulic acids (data not shown).

Results confirm the scavenging activity of synthesized polymers **3** and **4**. Analogously to what was observed with the lipid peroxidation, ferulate polymer was more active than the derivative containing lipoic moieties. The scavenging activities of both polymers were similar to those of the free lipoic and ferulic acids (data not shown). 

**Figure 3:**
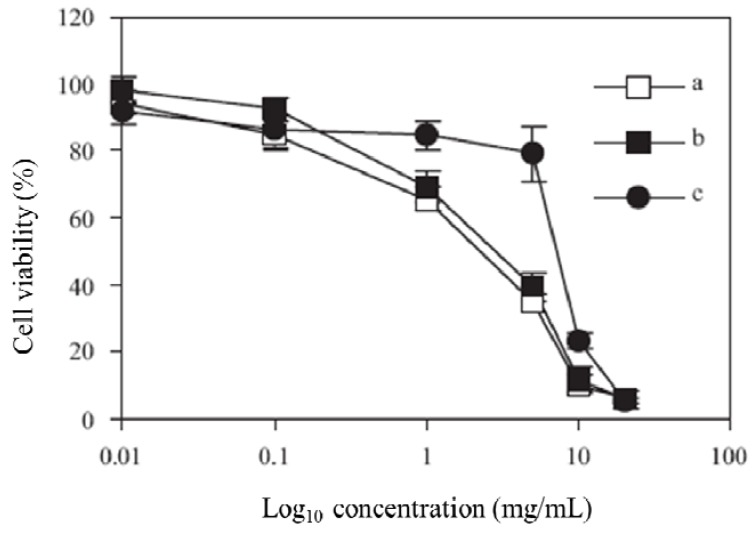
Viability of Caco-2 cells after incubation with polymer 1 (**a**), polymer 2 (**b**), and polymer 3 (**c**), at 37 °C for 4 h.

The MTT assay was performed to calculate IC_50_ values for the synthesized polymers. A graph of % cell viability *versus* concentration (Log_10_) is shown in Figure 3. The IC_50_ value, a concentration at which 50% of the cells are killed, is then calculated by taking 50% from the y axis. The IC_50_ values were 3.0, 3.8 and 7.6 mg/L for polymers **2**, **3** and **4**, respectively. 

## 3. Experimental

*Materials*. α-Lipoic acid (LA), *trans*-ferulic acid (FA), acryloyl chloride, methacryloyl chloride, dicyclohexylcarbodiimide (DCC), 1,4-D,L-dithiotreitol (DTT), 5-thio-2-nitrobenzoic acid (DTNB), Folin reagent, *N,N*-dimethylacrylamide (DMAA), vinylpyrrolidone (VP), L-cysteine, triethylamine, diphenylpicrylhydrazide (DPPH), methacrylic anhydride, 2,4,6-trinitrobenzensulfonic acid (TNBS), 1-hydroxybenzotriazole (HOBt), Triton X-100, MTT powder (≥97.5%), DMSO (99.5%), were supplied by Sigma-Aldrich (St. Louis, MO, USA). Acetonitrile, diethyl ether, methanol, ethanol, tetrahydrofuran (THF), *N,N*-dimethylformamide (DMF), toluene, chloroform, dichloromethane, acetone, water were obtained from Carlo Erba Reagenti (Milan, Italia). Modified Eagle’s Medium, Foetal Bovine Serum, sodium pyruvate (sterile), PBS (sterile), non-essential amino acids (sterile), L-glutamine (sterile), penicillin (sterile), streptomycin (sterile), trypsin (sterile), DPBS (sterile) were purchased from Life Sciences (Gibco BRL, Paisley, UK). Poly(ethylamine) (PEI, MW 25 kDa) was purchased from Polyscience, Inc (Warrington, PA, USA). Transwell^®^ polycarbonate membrane (sterile) were purchased from Corning Incorporated (Corning, NY, USA). 

*Measurements*. FT-IR spectra were measured on a Jasco 4200 spectrometer using KBr disks. Mass spectra were recorded on a Hewlett Packard GC-MS 5890 Series II. UV-VIS spectra were measured using a V-530 JASCO spectrophotometer at 25 °C using quartz cells of 1 cm path length.

*Cell culture*. Caco-2 cells at passage 8 were purchased from the ATCC (American Tissue Culture Collection, Manassas, VA, USA). HT29 cells (passage 121–128) were also obtained from the ATCC. Cells were grown and sub-cultured as previously described [38]. Caco-2 cells were maintained with MEM supplemented with FBS (10% w/v), sodium pyruvate (1 mM), non-essential amino acid (1% w/v), L-glutamine (1% w/v), penicillin (100 μg/mL) and streptomycin (100 μg/mL) at 5% CO_2_, 95% O_2_ at 37 °C.

*Synthesis of ethanolamine hydrochloride.* Gaseous hydrochloric acid, obtained by dripping a 37% HCl solution into 98% sulfuric acid was bubbled into a flask containing ethanolamine (2 mL) in ethyl ether (300 mL) under magnetic stirring until the formation of ethanolamine hydrochloride as a white precipitate was complete. The suspension was filtered and the obtained hydrochloride purified by recrystallization from ethanol. The reaction was highly exothermic and immediate.

*Synthesis of 2-aminoethylmetacrylate hydrochloride* (**1**). Ethanolamine hydrochloride (10 g, 103 mmol), methacryloyl chloride (15.38 mL, 0.148 mol) and hydroquinone (0.077g, 7 × 10^−3^ mol) were stirred for 1 hat 93–95 °C under N_2_. Hydrochloric acid formed was removed by passage through an alkaline bath. The obtained solution, viscous and yellow-brown coloured, was stirred for 2 h at 70–75 °C, then, cooled at 40 °C and THF (23 mL) added before cooled *n*-pentane (100 mL). The white creamy precipitate obtained was isolated by filtration, washed with *n*-pentane and dried at reduced pressure. The obtained product was purified though recrystallization from acetonitrile. Yield 70%. Compound (**1**) is a commercial product and the analytical results are analogous to literature data [39].

*Synthesis of N,N-dimethylacrylamide and 2-aminoethylmethacrylate based polymer* **2**. 2-aminoethylmethacrilate hydrochloride (1 g, 60.4 mmol), *N,N*-dimethylacrylamide (1.197 g, 0.121mol), dry DMF (10 mL) and 3% (w/w) of azobisisobutyronitrile (AIBN) were mixed at room temperature under N_2_. After complete homogeneization, polymerization was carried out for about 24 h at 60 °C under stirring and the white precipitate obtained was filtered off and washed with diethyl ether. Product **2** was purified by dissolution/precipitation cycles with solvent/non-solvent mixtures of distilled H_2_O and acetone. Finally, the polymer was treated with a NaOH solution (pH = 9–10), dialyzed, then lyophilized (conversion 91%); IR ν (cm^−1^) 1800, 1650, 1400, 1000). The amount of free amino groups present in the polymer was determined according to the Gaur Gupta method [35].

*Synthesis of α-lipoic acid containing polymer* **3**. DCC (413 mg, 2 mmol) was dissolved in dry CH_2_Cl_2_ (5 mL) under N_2_. The solution was cooled at −20 °C, then lipoic acid (309 mg, 1.5 mmol) was added, along with **2** (500 mg, 1.4 mmol), 1-hydroxybenzotriazole (HOBt, 270 mg, 2 mmol), and NEt_3_ (202 mg, 2 mmol) dissolved in dry CH_2_Cl_2_ (10 mL). The polymer obtained is insoluble or poorly soluble in most polar and apolar solvents. The product was purified by filtration and washing with diethyl ether, CH_2_Cl_2_, methanol, ethanol, water, acetone and diethyl ether again. The polymer was dried under reduced pressure. Its amount of free amino groups was determined according to the Gaur Gupta method [35] and that of the lipoic acid groups through oxidation of thiols with the Ellman reagent [40]. IR: ν (cm^−1^): 3440, 2936, 1731, 1634.

*Synthesis of trans-ferulic acid containing polymer* **4**. DCC (413 mg, 2 mmol) was dissolved in anhydrous CH_2_Cl_2_ (5 mL) under N_2_. To this solution was added **2** (952 mg, 3.4 mmol), ferulic acid (330 mg, 1.7 mmoles) and HOBt (344 mg, 2.55 mmol) in anhydrous CH_2_Cl_2_ (7 mL) at room temperature. After 24 h the product **4** was purified by repeated cycles of dissolution/precipitation in the solvent/non-solvent pair DMF and Et_2_O. The polymer was a yellow coloured powder. It was dried under reduced pressure (conversion 67%). Its amount of phenolic groups was evaluated by the method of Folin [36]. IR: ν (cm^−1^): 3440, 3334, 3100, 3000, 2927, 1726, 1696.

*Molecular weight analyses*: The molecular weight of polymers **2**, **3** and **4** was determined by multi-angle laser light scattering and gel permeation chromatography (GPC/MALLS, MiniDawn, Wyatt, CA, USA, equipped with a 20 mV semiconductor diode laser, vertically polarised, λ = 320 nm). The mobile phase was THF at a flow rate of 1 mL/min. About 200 µL samples were injected at a loading concentration of 1 mg·mL^−1^ using a Waters 717 plus autosampler and all determinations were carried out at room temperature. Refractive index increments (d*n*/d*c*) of polymers solutions in THF were measured with a Waters 2410 refractive index detector (λ = 850 nm) and data was processed using DNDC for Windows 5.10 software.

*Preparation of microsomal suspension*: Liver microsomes were prepared from Wistar rats by tissue homogenisation with 5 volumes of ice-cold, 0.25 M sucrose, containing 5 mM HEPES, 0.5 mM EDTA, at pH 7.5, in a Potter-Elvehjem homogeniser [41]. Microsomal membranes were isolated by removal of the nuclear fraction at 8,000 *g* for 10 min and by removal of the mitochondrial fraction at 18,000 *g* for 10 min. The microsomal fraction was sedimented at 105,000 *g* for 60 min, and the fraction was washed once in 0.15 M KCl and collected again at 105,000 *g* for 30 min [42]. The membranes, suspended in 0.1 M potassium phosphate buffer, at pH 7.5, were stored at −80 °C. Microsomal proteins were determined by the Bio-Rad method [43].

*Addition of lipoic, ferulic acids and the respective polymers to microsomes*: Aliquots of lipoic acid, ferulic acid and polymers **3** and **4** respectively, equal to 1 mg/mL, were added to the microsomes to obtain known antioxidant concentrations. Distilled water in the same amount was added to control microsomes. The microsomes were gently suspended using a Dounce homogeniser, and then the suspensions were incubated at 37 °C in a shaking bath under air in the dark.

*Malondialdehyde formation*: Malondialdehyde (MDA) was extracted and analyzed as indicated [44]. Briefly, aliquots of microsomal suspension (1 mL, 0.5 mg of proteins) were mixed with 0.5% TCA (3 mL) and TBA solution (0.5 mL, two parts 0.4% TBA in 0.2 M HCl and one part distilled water) and 0.2% BHT in 95% ethanol (0.07 mL). Samples were then incubated in a 90 °C bath for 45 min. After incubation, the TBA-MDA complex was extracted with isobutyl alcohol (3 mL). The absorbance of the extracts was measured by the use of UV spectrophotometry at 535 nm, and the results were expressed as percentage of MDA inhibition.

*Evaluation of the antiradical activity using DPPH test*: The ability of polymers **2** and **3** to act as radical scavengers was considered. Their radical scavenging ability was assessed through the reaction with stable DPPH radicals, using the methodology of [45]. DPPH typically extracts a proton to form the reduced DPPH [46]. Briefly, in an ethanol solution of DPPH radical (final concentration 8 × 10^−5^ M), polymers were added, and their concentrations were 5, 10, 20, and 40 mg/mL. The reaction mixtures were stirred vigorously and then kept in the dark for 30 min. Their absorbances were measured in 1 cm cuvettes using a UV-Vis spectrophotometer (V-530 JASCO) at 517 nm against a blank, in which DPPH was absent. All tests were run in triplicate and averaged. 

*Biocompatibility evaluation by MTT assay*: In order to investigate the toxicity of polymers **2**, **3** and **4**, Caco-2 cells were cultured in 96-well plates at a cell density of 5 × 10^5^ cells/well; every column receiving different dilutions prepared from a stock suspension (10 mg/mL) of polymer. After that, cell cultures were incubated (37 °C, 5% humidity, 95% CO_2_) for 4 h where the content was then discarded, washed with DPBS and replaced with fresh medium and incubated (37 °C, 5% humidity, 95% CO_2_) for 24 h after which Triton (40 μL) was added to the negative control and incubated (37 °C, 5% humidity, 95% CO_2_) for 1 h. The content was discarded and the tetrazolium compound MTT (200 μL) was added to each well and incubated (37 °C, 5% humidity, 95% CO_2_) for 30 min. The content was again discarded and DMSO (200 μL) was added to each well with 15 min of incubation (37 °C, 5% humidity, 95% CO_2_) and the plates were read by plate reader (EL_X_ 808, bio-TEK Instruments Inc., Vermont, VA, USA) to determine the IC_50_ values of each polymer by plotting the % cell viability *vs*. log_10_ concentration.

## 4. Conclusions

In this work, we prepared biocompatible polymers with antioxidant activity using a synthetic route in which we initially obtained, by radical copolymerization, a polymer possessing free amino groups in the side chain and the reactivity of these groups was used for the subsequent inclusion of two well-known and powerful antioxidants: α-lipoic acid and *trans*-ferulic acid.

We determined the amount of non-reacted amino groups (moles of amino groups per gram of polymer) for the thus obtained polymers by the Gaur-Gupta method. For polymers bearing in the side chain α-lipoic acid residues the degree of functionalization (moles of lipoic groups/gram of polymer) was evaluated by thiol groups determination with the Ellman reagent after reduction with DTT. For polymers bearing ferulic residues in the side chain the degree of functionalization was determined by phenolic groups determination by the Folin method.

In all prepared systems the antioxidant effects were evaluated by two different methods. The activity to directly block radical species (scavenger activity) was evaluated through spectophotometric analysis by measuring the decrease in concentration of a radical (DPPH) chromophore. The ability to inhibit lipid peroxidation was spectrophotometrically assessed by following the formation of malondialdehyde produced in liver rat microsomal membranes and induced by a pro-oxidant agent (*tert*-BuOOH). The results obtained with both tests have shown that the polymeric systems designed displayed a concentration-dependent antioxidant activity comparable to that of commercial lipoic and ferulic acids. Finally, it is noteworthy that these polymers are biocompatible with Caco-2 cells used as an example of absorptive epithelial cells. The thus obtained antioxidant polymers could be used in the biomedical and pharmaceutical fields and could substantially reduce free radical damage.
